# Soluble Saccharated Oxide of Iron as an Antidote to Arsenic

**Published:** 1870-12

**Authors:** 


### Soluble Saccharated Oxide of Iron as an Antidote to Arsenic.

Dr. Kohler, of Halle, remarks that the long-known antidote, *hydrated oxide* of iron has many practical inconveniences. The preparation now recommended only differs from the latter in containing a larger proportion of water [as hydrate.] Kohler used it with remarkable success in the case of a young man who had swallowed thirty or forty grains, or more, of arsenic. He comes to the following general conclusions about the new therapeutic agent: 1. That it precipitates arsenious acid from solution in the form of insoluble arseniate. 2. That on chemical grounds it should be justly substituted for the ordinary hydrated oxide as an antidote. 3. The experiments on animals fully bear out its practical efficacy. 4. That, while in other forms of metallic poisoning [especially with common sublimate] mechanical antidotes like albumen, etc., are useful, the latter treatment is only a hinderance to the efficient application of the oxide of iron in arsenical poisoning. 5. That the iron-treatment should *not* be accompanied by the use of neutral purgative salts, otherwise the antidotal combinations may be interfered with. 6. Since Schroff has proved that the arseniate of iron itself is always absorbed in minute quantities, emetics should be administered as soon as the antidotal combination of the iron with the arsenic may be supposed to have taken place. 7. As to the quantity of saccharated oxide of iron required to neutralize a given quantity of arsenic, it appears that about ten or twelve parts of the oxide should be administered for every one part of arsenic believed to have been swallowed.—*Berlin Klin. Wochensch.*—*N. Y. Med. Jour.*

